# Natural Occurrence of Ochratoxin A in Blood and Milk Samples from Jennies and Their Foals after Delivery

**DOI:** 10.3390/toxins12120758

**Published:** 2020-12-01

**Authors:** Vincenzo Lippolis, Shafaq Asif, Michelangelo Pascale, Salvatore Cervellieri, Erminia Mancini, Angelo Peli, Ippolito De Amicis, Domenico Robbe, Fiorenza Minervini

**Affiliations:** 1 Institute of Sciences of Food Production (ISPA), National Research Council of Italy (CNR), 70125 Bari, Italy; vincenzo.lippolis@ispa.cnr.it (V.L.); sasif@unite.it (S.A.); michelangelo.pascale@ispa.cnr.it (M.P.); salvatore.cervellieri@ispa.cnr.it (S.C.); erminia.mancini@ispa.cnr.it (E.M.); 2 Faculty of Veterinary Medicine, University of Teramo, 64100 Teramo, Italy; ideamicis@unite.it (I.D.A.); drobbe@unite.it (D.R.); 3 Department of Veterinary Medical Sciences, Alma Mater Studiorum—University of Bologna, Ozzano dell’Emilia, 40064 Bologna, Italy; angelo.peli@unibo.it

**Keywords:** jennies, ochratoxin A, pregnancy, milk blood, placental transfer, foals

## Abstract

An assessment of the natural ochratoxin A (OTA) exposure of seven Martina Franca jennies was carried out by analyzing blood and milk samples collected close to and after delivery. A total of 41 and 34 blood samples were collected from jennies and foals, respectively, and analyzed by ELISA. A total of 33 milk samples were collected from jennies and analyzed by the HPLC/FLD method based on IAC clean-up. Furthermore, 53 feed samples were collected from January to September and analyzed by a reference method (AOAC Official Method No. 2000.03) for OTA content. Feed samples showed OTA levels up to 2.7 ng/g with an incidence of 32%, while the OTA incidence rate in jennies’ blood samples was 73%, with a median value of 97 ng/L and concentrations ranging from <LOD to 6000 ng/L. A seasonal effect on OTA levels in positive blood samples was observed, with increases in the 53% of the positive ones from April to June. Concerning foals, the incidence rate of blood samples was 50%, with a median value of 52 ng/L, and concentrations ranged from <LOD to 4034 ng/L. The incidence of milk samples was 36%, with levels ranging from <LOD to 82 ng/L. In conclusion, the results showed a natural exposure of jennies and foals to OTA, and its presence in jenny milk could pose a risk for human newborns, considering its well-known nutritional and health properties.

## 1. Introduction

Ochratoxin A (OTA) is a major mycotoxin produced by several species of fungi, including *Aspergillus ochraceus*, *A. carbonarius*, *A. niger*, and *Penicillium verrucosum* [1]. Contamination generally occurs because of suboptimal drying practices and poor storage conditions of commodities [2]. OTA has been reported as a ubiquitous natural contaminant of food and feed [3,4,5]. Cereals, such as oat, wheat, barley, and their by-products, such as bran, being widely used as feed raw materials are the major cause of OTA exposure to animals. The intake of feed contaminated by OTA represents a potential risk for animal health and a food safety issue mainly due to the transfer of the toxin through the food chain to humans [6]. Interest in OTA increased when OTA was classified by the International Agency for Research on Cancer as a possible human carcinogen (Group 2B), based on evidence of carcinogenicity in experimental animal studies [1]. Ochratoxin A has a potent toxicity, and its nephrotoxic, hepatotoxic, teratogenic, carcinogenic, and immunosuppressive effects have been demonstrated in several mammalian species [7]. The use of OTA-contaminated feed during a long rearing period involves serious risk as a result of reduced feed efficiency, with decreased growth and weakening of the immune system [8]. Monogastric (without rumen) species, for example humans, swine, and ruminants (with immature/partially functioning rumen), are at higher risk, lacking appropriate ruminal microbiota and microbes for the degradation of OTA to less toxic compounds [9]; consequently, regular exposure to OTA can be a threat to monogastric species. Chronic human exposure to low levels of OTA occurring frequently in foods is more alarming than acute exposure to higher doses [3]. In addition, human fetuses and newborns are exposed to OTA through transplacental transfer (especially in early gestation), inducing possible reprotoxicity, embryotoxicity, and teratogenicity, or through OTA-contaminated human breast milk or infant formula [10,11]. Several studies have demonstrated transplacental transfer of OTA in swine, although contrasting reports have been published showing no residues in piglets of sows fed diets containing OTA through gestation and no placental transfer after ingestion of OTA by a pregnant sow [12,13]. In contrast, some authors found OTA transmission to piglets in uteruses with blood concentrations ranging from 0.075 to 0.12 ng/mL [5]. Minervini et al. [14] reported OTA exposure of mares and transplacental transfer with consequent foal exposure. The mean ratio of OTA maternal blood/fetal blood was 1.96 ± 0.94, probably due to its active transport across the placenta, as a consequence of OTA chemical characteristics (such as the similarity between the chemical structures of OTA and phenylalanine, low molar mass, lipophilic character, and serum binding property) [14]. Concerning jennies, their feed typically consists simply of grass, hay, and minerals, excluding the possibility of mycotoxins exposure. In the case of professional jenny breeding farms, and for lactating animals, cereals and cereal-based pellets are added to the diet with consequent carry-over of mycotoxins into milk that is considered to be similar to human breast milk in nutrient composition, and it represents a valid alternative in the infant’s diet in terms of nutritional adequacy for subjects affected by allergy to cow milk proteins [15]. To date, as far as we know, official information about the production of donkey milk and its human consumption in Italy and Europe is not available [16]. Eight major Italian farms, with an average consistency of about 100 donkeys, declared an average annual sale of donkey’s milk for food purposes of about 400,000 L [17]. Around 40% of Italian producers sell donkey milk exclusively for human nutrition, whereas 60% sell the milk for both human consumption and processing into cosmetics [16].

However, currently, the consumers’ interest for this product is increasing, and it is gaining importance and international acceptance [16]. Indeed, it is rich in lactose and whey proteins, contributing to the intestinal absorption of calcium essential for bone mineralization. The presence of endogenous bioactive compounds increases some other alleged health benefits, such as antibacterial activity, the stimulation of immune system, the prevention of inflammatory diseases, and antiaging properties [15]. To our best knowledge, only one study has been reported in the literature showing no OTA occurrence in jenny milk analyzed by ELISA [18].

Presently, no data are available on jenny OTA exposure and the relevant transplacental transfer. The purpose of this study was to assess the natural exposure to OTA of jennies and their foals, reared extensively in open stabling, both by performing analysis of feed, blood, and milk samples from jennies and their foals close to and three months after delivery.

## 2. Results

### 2.1. Animals

During the entire period of study, jennies did not show any systemic symptoms, such as hyperthermia, congestion, respiratory symptoms, or gastrointestinal disorders (e.g., malaise, diarrhea, abdominal colic pain, lack of appetite, and sialorrhea). In addition, the gestation was completed without pregnancy pathologies or interruptions. All jennies had a normal course of pregnancy and complied with the criteria for normal parturition. All foals (three females and four males) were born at term and by spontaneous eutocic parturition. All animal procedures performed in this study met the requirements of Italian law on the use of animals for experimental and other scientific purposes (Legislative Decree 26/2014, implementing Directive 2010/63/EU on the protection of animals used for scientific purposes), and the research protocol was approved by the Ministry of Health (authorization n 370/2020-PR, 24 April 2020).

### 2.2. OTA Contamination in Feed

Table 1 shows the OTA levels found in feed samples that were grouped into three major groups according to the level of OTA contamination. The incidence of positive feed samples was very low (32%); OTA concentrations found in feed samples ranged from 0.3 to 2.7 ng/g, were far below the guidance OTA values in feed materials (cereals and cereal-based products), and composed feeds for pigs, poultry, cats, and dogs reported by the Commission Recommendation 2006/576/EC [18].

The analysis of each cereal component present in the feed showed similarly low OTA levels (0.15–0.18 ng/g) in bran, corn flake, and soy, whereas barley and oat were uncontaminated (detection limit of 0.1 ng/g). Toxin concentrations were far below the guidance values of OTA in feed materials reported by the Commission Recommendation 2006/576/EC [19]. No seasonal pattern of OTA contamination in feed samples was observed.

### 2.3. OTA Occurrence in Blood Samples Collected from the Jennies.

As observed in Table 2, the OTA incidence rate of blood samples (with OTA levels higher than the detection limit) collected 15 days before the delivery and at the delivery was 71% and 86%, with OTA median levels of 78 and 97 ng/L, respectively. Interestingly, a high level of OTA was observed in May for Falaria jennies. This high level of OTA in the blood samples could be related to a seasonal effect that was confirmed by the OTA levels found in all blood samples collected during the entire study.

**Figure 1:**
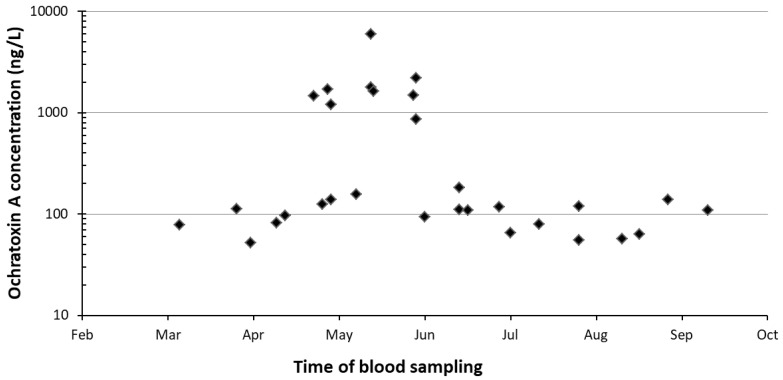
Seasonal distribution of contaminated blood samples collected from jennies 15 ± 5 days before pregnancy and three months after delivery.

In fact, as observed in Figure 1, a significant increase from 2 to 120 times in 53% of the positive ones collected from April to June was observed. 

After delivery, the mean incidence of positive blood samples was 73%, with a median value of 109 ng/L and OTA ranging from <LOD to 6000 ng/L (Table 3). No significant differences among OTA levels in blood samples collected from jennies were found.

### 2.4. OTA Contamination in Blood Samples from Foals

At the time of delivery, no OTA was recorded in blood samples collected from all foals, attesting no placental transfer (Table 2). As observed in Table 3, the total number of positive blood samples was 17, whereas the incidence rate was 50% in foals. The median value was 52 ng/L, with a range from <LOD to 4034 ng/L. No statistical differences in OTA levels in blood samples were found among foals. No relation between OTA levels in the blood samples of jennies and related foals was recorded, because animals were reared extensively in open stabling, a typical condition for donkeys that favors the suckling of milk even by different jennies.

### 2.5. OTA Contamination in Milk Samples

As observed in Table 3, the total number of positive milk samples was 12, whereas the incidence rate was 36%. The median value was 7.5 ng/L, and ranged from <LOD to 82 ng/L. In six jennies, incidence of OTA contamination ranged from 25% to 60%, with levels up to 82 ng/L. Only one jenny produced one milk sample with 41 ng/L of OTA, and another jenny did not excrete OTA by milk. Francisca and Antiqua were the only jennies with a higher value of incidence and consequent quantified OTA amount in milk, and this condition could be related to the period of delivery. 

**Figure 2:**
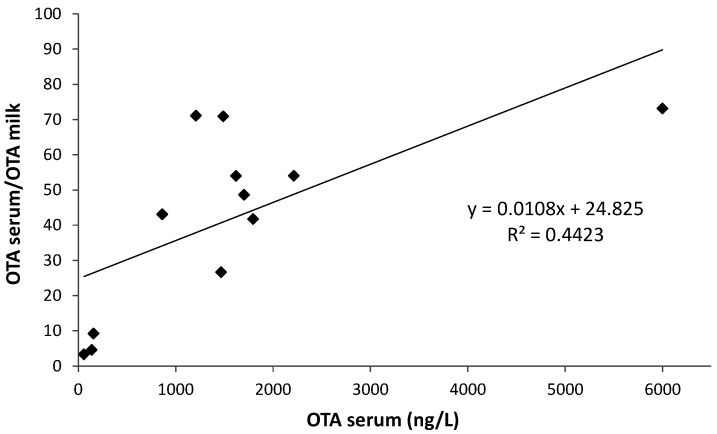
Relationship between the serum ochratoxin A (OTA) and serum/milk OTA ratio in samples collected from jennies.

As observed in Figure 2, when considering only the positive milk and blood samples, a significant linear correlation was observed between the OTA level in serum and the OTA serum/OTA milk concentration ratio (*r*^2^ = 0.442; *p* < 0.001).

## 3. Discussion

In contrast to in vivo toxicological studies, in which defined amounts of mycotoxins are administered and it is possible to determine their blood levels, this study was planned to monitor and evaluate the exposure of jennies to OTA under natural conditions, using non-artificially contaminated feed and keeping animals reared extensively in open stabling, as donkeys are normally bred. The natural exposure of jennies and their foals to OTA was assessed by analyzing feed samples and the relevant biomarker, i.e., OTA in blood and milk samples, in order to test the individual exposure of jennies and their foals and to check the safety of milk for minimizing hazards to human health. 

OTA contamination in the bulk of cereals is often randomly distributed in localized “hot spots”, with high variability, and this characteristic property of contamination may reflect the fact that OTA is typically produced during storage. Nonetheless, it is very difficult to determine the extent of mycotoxin contamination in feedstuffs because mycotoxins are unevenly distributed in feed, introducing a significant amount of sampling error into sample analyses.

According to the European Commission (EC) regulation, official control of the levels of OTA in lots of cereals and cereal products has to be performed by collecting incremental samples (up to 100 samples) to obtain a laboratory sample that should be representative of the entire lot. In order to overcome an underestimation of OTA contamination and to provide an accurate and precise OTA determination value, the slurry-mixing procedure is strictly necessary, as reported by Lippolis et al. [20]. This procedure was used in this study to reduce the variability related to the sampling of concentrated feed. The OTA levels found in a single component of the mixture and in the concentrate mixture were lower than the values laid down by the Commission Recommendation 2006/576/EC [19], equal to 250 mg/kg in the case of cereals and cereal-derived products, and for complementary and complete feedstuffs are 50 and 100 mg/kg for pigs and poultry, respectively. This paper confirmed that, although feed sampling was conducted according to European regulations and despite the use of the slurry, feed analysis is not an accurate approach to assess the exposure of animals to OTA. In fact, the incidence of OTA-contaminated feed samples was lower than the incidence of OTA in blood samples, validating the usefulness of biomarkers as a more accurate approach for assessing OTA exposure. Concerning blood samples from jennies, OTA levels did not affect the jennies’ health and pregnancy. In addition, the median value and the incidence of OTA levels in blood samples collected from jennies were lower than those found in horses (121.4 ng/L and 83%, respectively), probably due to diet composition [14]. In fact, the majority of horses (from 64% to 80%) fed with commercial feed, hay, and oats had less OTA in serum (mean value = 150 pg/mL) than horses fed with bran (mean value = 339 pg/mL) [14]. Instead, the jennies, notoriously more rustic animals than horses, have a diet mainly represented by hay with a supplement of feed during pregnancy and lactation. An increased OTA level in blood samples that was seasonally related was recorded, probably due to possible OTA contamination of the hay. As reported by Fink-Gremmels [6], the ensiled grass or hay may contain a complex mixture of mycotoxins, originating from a pre-harvest contamination by *Fusarium* spp. toxins, as well as from post-harvest contamination with toxins produced by fungal species that are common in silage during the storage, such as *Penicillium*, *Aspergillus*, *Monascus*, and *Trichoderma*, some of which are OTA producers [6]. Concerning foals, no placental transfer was observed, because the blood samples collected from umbilical cords after delivery did not contain OTA. These results were in disagreement with data reported by Minervini et al. [14] on the exposure of foals after the delivery of mares, consequent to the placental transfer, with higher blood OTA concentrations (from 69.5 to 252.6 ng/L). The ratio between the mare’s and foal’s OTA serum levels was variable, and no correlation (*r* = −0.07) was found. The absence of a correlation between OTA levels in umbilical and mare serum samples was in agreement with the results reported in pigs after in vivo exposure [21], and could be explained by several factors, such as exposure time (gestation period and related placental vascularization) and/or placental structure. Concerning the gestation period, during the early stage of pregnancy, OTA or its metabolites could pass through the placenta into the fetal circulation and accumulate in the fetal tissues, exerting developmental toxicity. Concerning placental structure, swine and equine placenta are characterized by a chorionic epithelium, which could prevent or interfere with the access of the toxin to the umbilical blood in vivo and consequent OTA fetal uptake, strictly related also to the developmental stage of the placenta. The disagreement of placental transfer between mares and jennies could be related to different anatomical structure of the placenta in both animal species; in fact, as reported by Veronesi et al. [22], although strong morphological similarities exist between the allantochorion of the horse and jenny, the jenny develops more complex microcotyledons, as judged stereologically, and exhibits a lower placental efficiency. The OTA exposure of foals after delivery should be realized through milk, as confirmed by Francisca and relevant foal, showing the highest levels of OTA both in the blood of jenny and foal and in milk samples. As observed in Table S1 (Supplementary Material), all donkeys that gave birth or nursed in the period between April and May showed high concentrations of OTA in their blood and, consequently, simultaneous presence of OTA in milk and in foal blood. An exception is given by the jenny Falaria (delivery date May 31) showing high levels of OTA in blood and the presence of OTA in milk at delivery, while the foal showed an absence in the blood. For the other jennies, it was not possible to find any relationship, probably due to the fact that the animals were reared extensively in open stabling. A positive relationship between the serum OTA level and serum/milk OTA concentration ratio was found in this study, and was in agreement with data found in humans [10]. The same pattern of OTA excretion found in women and jennies indicated that, at an increasing OTA blood level, the carry-over to milk rises less than proportionally, suggesting a decrease in the efficiency of carry-over and probably a saturation of the transport system [10]. The levels of OTA found in jennies’ milk were 10 times lower than those reported in women’s milk samples at the same incidence [10]. On the contrary, similar levels, but with higher incidence (almost 60%), in human milk samples was reported [11]. Recently, the EFSA Panel on Contaminants in the Food Chain (CONTAM) reported a study from European countries with breast milk OTA levels ranging between 10 and 400 ng/L [23]. The major OTA contamination/incidence found in human milk samples should be due to both different sources of OTA exposure (different foods suspected of contamination) and to the high half-life (35 days) in humans. Presently, no data are available concerning the OTA half-life in equine species. The occurrence of OTA in milk samples collected from jennies in this study was in disagreement with the results reported by Gross et al. [18], probably due to the different analytical determination used (ELISA with respect to HPLC) and to the low number of milk samples (*n* = 6). In our study, continuous monitoring of donkey milk has allowed to show the presence of OTA in the blood and milk probably due to a batch of hay of poor quality and containing OTA. The seasonal effect observed on OTA presence in milk samples should indicate the need of continuous monitoring of OTA levels in donkey milk in order to guarantee the safety of this food intended for infant consumption. Further studies should be carried out in order to confirm the contamination of donkey milk with OTA, which could pose a real health risk to newborns.

## 4. Materials and Methods 

### 4.1. Chemicals

Acetonitrile and methanol (both HPLC grade) were purchased from Carlo Erba Reagents (Milan, Italy). Dichloromethane and hydrochloric acid (both HPLC grade) were purchased from Mallinckrodt Baker (Milan, Italy). Ultrapure water was produced by a Millipore Milli-Q system (Millipore, Bedford, MA). Ochratoxin A (OTA), sodium chloride (NaCl, ACS grade), sodium hydrogen carbonate (NaHCO_3_, ACS grade), Tween 20, and acetic acid were purchased from Sigma-Aldrich (Milan, Italy). OTA immunoaffinity columns (OchraTest™) were obtained from VICAM, A Waters Business (Milford, MA). Glass microfiber filters (GF/A) and paper filters (No. 4) were purchased from Whatman (Maidstone, UK). OTA immunoaffinity columns (OchraPrep^®^) and ELISA test kits (RIDASCREEN^®^ Ochratoxin A 30/15) were purchased from R-Biopharm (R-Biopharm AG, Darmstadt, Germany).

### 4.2. Animals and Clinical Data

The study was conducted from March to September 2020. Seven pregnant Martina Franca jennies, with ranges of age 5–11 years and weight 370–510 kg, were enrolled at the animal farm at the University of Teramo, Italy. All pregnant jennies were in good health and body conditions. The seven jennies were grouped in small herds and were kept in outdoor paddocks. Jennies were fed daily ad libitum with standard hay supplemented with concentrate feed (i.e., a mix of cereals) at different amounts in relation to physiological conditions two months before and three months after the delivery. The choice of this period has been defined due to the increasing of energy requirements and nutritional needs in the final stage of pregnancy and during lactation. Foals were fed with jenny milk during the entire period of the study.

### 4.3. Feed, Blood, and Milk Sampling

Fantini S.r.l. Italy (a feed mill Company) prepared a cereal mixture (concentrated feed) composed of several cereals at different percentages. The complete feed for the jennies comprised a mixture of cereal components: oats (35%), flacked barley (35%), flacked corn (10%), bran (15%), soy bean (4.5%), and mineral vitamin components (0.5%). Concentrated feeds were packed in 25 kg bags and stored at room temperature.

Sampling of feed, blood, and milk samples was performed during the interval from January to September at the animal farm that belongs to the Faculty of Veterinary Medicine, University of Teramo, Italy. Specifically, blood and milk samples were simultaneously collected on the scheduled day.

The sampling of concentrated feed samples was carried out according to the sampling procedures of the EU Regulation (CE) N. 152/2009 at the opening of each bag [20]. A total of 53 feed samples (about 500 g each) were collected and stored at room temperature. Before the preparation of concentrated feed, each component of the mixture was collected following the sampling protocol, as previously described [24].

Concerning blood sampling (*n* = 41), the first and second samples were collected using stored aliquots that were obtained from the routine sampling taken from jennies for clinical purposes, respectively, 15 ± 5 days before the expected date of delivery and at the delivery. After delivery, due to extensive farming in open stables, the blood and milk sampling was carried out for two or three months, respecting a periodicity of 15 ± 5 days. The total number of blood samples tested after delivery was 34. With respect to foals, blood samples (*n* = 34) were collected at the delivery from the umbilical cord and every 15 ± 5 days during the subsequent two or three months. Sampling dates are reported in Table S1 in Supplementary Materials.

Blood samples (2–5 mL per jenny and the respective foal) were collected in individual sampling tubes and stored at −20 °C until analysis. At the time of delivery and after delivery, every 15 ± 5 days for a consecutive two or three months, 2–3 mL milk samples (*n* = 33) were collected from each jenny. Milk samples were stored at −20 °C until analysis.

### 4.4. OTA Extraction and Purification

#### 4.4.1. Feed Samples

Feed samples and relevant cereal components were comminuted/homogenized by mixing with the Ultra Turrax IKA T50 mixer (IKA Werke GmbH & Co. KG., Staufen, Germany) after adding three times their weight of water (water/matrix ratio of 3:1). After 10 min of mixing, slurry samples were stored at −20 °C until HPLC analysis. For the determination of OTA, homogenized feed samples were extracted following the AOAC Official Method No. 2000.03, with minor modifications [25]. The extraction procedure for 100 g slurry-mixing portions (equivalent to 25 g of feed) was carried out by shaking (60 min) using 112.5 mL of acetonitrile in order to obtain the same ratio among the sample, acetonitrile, and water, as in the official method procedure. The extraction mixture was filtered through filter paper (Whatman No. 4) to remove particulate matter. A 10 mL volume of filtered extract was diluted with 40 mL of distilled water, then mixed and filtered through a glass microfiber filter (Whatman GF/A); 20 mL of the diluted extract was loaded onto a OchraTest™ immunoaffinity column and passed through the column at a flow rate of about 1 drop/s. The immunoaffinity column was washed with 10 mL of wash buffer (2.5% NaCl, 0.5% NaHCO_3_, 0.01% Tween 20) and 10 mL of distilled water at a flow rate of 1–2 drops/s. OTA was eluted with 1.5 mL of methanol and collected in a silanized vial. The eluted extract was dried under a nitrogen stream at about 50 °C, and reconstituted with 500 µL of HPLC mobile phase [acetonitrile-water-acetic acid (99 + 99 + 2, *v/v/v*)].

#### 4.4.2. Serum Samples

OTA determination in blood samples was performed with ELISA test kits (RIDASCREEN^®^ Ochratoxin A 30/15) according to the protocol provided by the manufacturer (R-Biopharm AG, Darmstadt, Germany), with minor modifications. The extraction procedure for 2 mL of thawed and vortexed serum samples was carried out by shaking (5 min) using 2.5 mL of 1 N HCl and 4 mL of dichloromethane. Shaking was followed by centrifugation for 15 min at 3500 g and 15 °C. The upper aqueous layer was removed. The dichloromethane layer was filtered by filter paper (Whatman No. 4), and 2 mL of filtrate was extracted with 2 mL of sodium hydrogen carbonate buffer (0.13 M, pH 8.1). After shaking (5 min), the sample was centrifuged for 5 min at 3500 g and 15 °C. The step of extraction with sodium hydrogen carbonate buffer was repeated twice. The double layers of sodium hydrogen carbonate buffer were combined and extracted with 2 mL of dichloromethane and 0.75 mL of 1 N HCl. After shaking (10 min) and centrifugation (5 min at 3500 g and 15 °C), the buffer layer was discarded. The extract was dried under a nitrogen stream at about 50 °C, and reconstituted with 1 mL of sodium hydrogen carbonate buffer. Samples were kept at 2–8 °C until ELISA analysis. A subset of randomly selected samples (*n* = 17) was also purified using the immunoaffinity column, according to Curtui et al. [26], with minor modifications, and analyzed by HPLC to confirm ELISA results (Table S2, Supplementary Material). In particular, 840 µL of the reconstituted sample was diluted with 4.2 mL of sodium hydrogen carbonate buffer and mixed, and 4 mL of the diluted sample was loaded onto an OchraTest™ immunoaffinity column and passed through the column at a flow rate of about 1 drop/s. The immunoaffinity column was washed with 10 mL of wash buffer (2.5% NaCl, 0.5% NaHCO_3_, 0.01% Tween 20) and 10 mL of distilled water at a flow rate of 1–2 drops/s. OTA was eluted with 1.5 mL of methanol and collected in a silanized vial. The eluted extract was dried under a nitrogen stream at about 50 °C, and reconstituted with 250 µL of HPLC mobile phase [acetonitrile-water-acetic acid (99 + 99 + 2, *v/v/v*)].

#### 4.4.3. Milk Samples

OTA determination in milk samples was performed by using the HPLC method based IAC purification reported by Bascarán et al. [27], with some modifications. Whole milk samples (2 mL) were loaded onto an OchraPrep^®^ immunoaffinity column and passed through the column at a flow rate of about 1 drop/s. The immunoaffinity column was washed twice with 10 mL of distilled water at a flow rate of 1–2 drops/s. OTA was eluted with 3 mL of methanol and collected in a silanized vial. The eluted extract was dried under a nitrogen stream at about 50 °C, and reconstituted with 250 µL of HPLC mobile phase [acetonitrile–water–acetic acid (99 + 99 + 2, *v/v/v*)].

### 4.5. HPLC Analysis: OTA Standard Solutions and Recovery Experiments

An aliquot (100 µL) of reconstituted feed, serum, or milk extract (corresponding to 0.107 g, 0.32 mL, and 0.8 mL of feed, serum, and milk, respectively) was injected into the chromatographic system. HPLC analyses were carried out using an Agilent 1260 Series chromatographic system (Agilent Technologies, Palo Alto, CA, USA) equipped with a fluorometric detector (model G1321B, λ_ex_ = 333 nm, λ_em_ = 460 nm). The analytical column was a Zorbax SB-C18 (5 μm, 4.6 × 150 mm; Agilent Technologies), preceded by a 0.5 µm Rheodyne guard filter (IDEX Health & Science, Wertheim-Mondfeld, Germany); the mobile phase was acetonitrile-water-acetic acid (99 + 99 + 2, *v/v/v*), and a flow rate of 1 mL/min was used. OTA concentrations are reported as ng/g, ng/L, and ng/L for feed, serum, and milk samples, respectively. The detection limits (*S*/*N* 3:1) of the methods were 0.1 ng/g, 50 ng/L, and 15 ng/L for feed, serum, and milk, respectively. Similarly, quantification limits (*S*/*N* 10:1) of 0.33 ng/g, 166.7 ng/L, and 50 ng/L were calculated for feed, serum, and milk, respectively.

OTA stock solution was prepared by dissolving a solid commercial toxin in toluene/acetic acid 99:1 (*v*/*v*) at a concentration of 1 mg/mL. An OTA standard solution in methanol at the concentration of 10 μg/mL was prepared and spectrophotometrically tested (ε = 6330 cm^2^/mmol, at λ = 332 nm in methanol). For spiking purposes in recovery experiments and for the preparation of standard solutions for HPLC, a solution of OTA was prepared in methanol at a concentration of 250 ng/mL.

Concerning feed, recovery experiments were performed by spiking three feed samples with OTA at a level of 5 ng/g, showing an average recovery of 108%, with a relative standard deviation of 5%. Concerning serum, recovery experiments were performed by spiking three serum samples with OTA at a level of 500 ng/L. Spiked samples were extracted and analyzed by ELISA, showing an average recovery (mean) of 104%, with a relative standard deviation of 25%. Concerning milk, recovery experiments were performed in triplicate by spiking milk samples with OTA at a level of 100 ng/L. Spiked samples were extracted and analyzed by the HPLC method, showing an average recovery (mean) of 79%, with a relative standard deviation of 10%.

### 4.6. Statistical Analysis

Statistical analysis was performed using the SigmaPlot™ software v.12 (Systat Software, Inc., SigmaPlot for Windows). Concerning blood and milk samples, a value corresponding to half the detection limit was assigned to all values below the detection limit [28]. Before testing for group differences, normality of the data distribution was assessed in OTA contamination of pooled serum samples collected for each jenny using the Shapiro–Wilk test. Since the data were not normally distributed, the median of the OTA concentration in serum samples was reported.

The comparison among pooled serum data of each jenny or foal was performed by one-way ANOVA and the Kruskal–Wallis non-parametric test. The relation between OTA levels in serum samples from jennies and the serum/milk OTA ratio was performed using linear regression analysis.

## Figures and Tables

**Table 1:** Incidence (%) and concentrations (ng/g) of Ochratoxin A (OTA) contaminations in feed samples for jennies assessed by HPLC analysis.

Feed Samples	Number of Feed Samples with OTA Concentration		
Contamination ranges (ng/g)	<0.3 ^a^	From 0.3 to 1	From 1 to 3
Number of samples	36	14	3
Incidence of contamination	68%	26%	6%
Range of OTA level (ng/g)	- ^b^	0.3–0.7	1.4–2.7

^a^ Limit of quantification (LOQ) = 0.3 ng/g; ^b^–not assessable.

**Table 2:** Ochratoxin A (OTA) levels in blood collected from jennies and their foals 15 ± 5 days before pregnancy and at delivery.

Jenny Name and Relevant Foal	[OTA] (ng/L) before Delivery	[OTA] (ng/L) at Delivery
Adelaide	<LOD ^a^ (March)	52 (April)
Foal	- ^b^	<LOD
Francisca	112 (March)	97 (April)
Foal	- ^b^	<LOD
Gaia	93 (June)	109 (June)
Foal	- ^b^	<LOD
Etiopia	125 (April)	139 (May)
Foal	- ^b^	<LOD
Antiqua	78 (March)	<LOD (April)
Foal	- ^b^	< LOD
Falaria	<LOD (May)	2215 (May)
Foal	- ^b^	<LOD
Eritrea	65 (July)	79 (July)
Foal	- ^b^	<LOD

^a^ Limit of detection (LOD) = 50 ng/L; in parenthesis is the month of sampling; ^b^–not assessable.

**Table 3:** Ochratoxin A (OTA) levels in blood samples collected from jennies and foals and milk samples after delivery.

Jenny Name	Jennies’ Blood Samples		Milk Samples		Foals’ Blood Samples	
Jennies	Positive/Total Samples	Median	Positive/Total Samples	Median	Positive/Total Samples	Median
	(%)	(Range, ng/L)	(%)	(Range, ng/L)	(%)	(Range, ng/L)
Adelaide	4/6	53.5	2/5	<LOD ^b^	3/5	109
	(67%)	(<LOD ^a^–1796)	(40%)	(<LOD ^b^–43)	(60%)	(<LOD ^a^–4034)
Francisca	4/5	1489	3/5	21	4/5	144
	(80%)	(<LOD ^a^–6000)	(60%)	(<LOD ^b^–82)	(80%)	(<LOD ^a^–594)
Gaia	2/5	<LOD ^a^	0/5	<LOD ^b^	0/5	<LOD ^a^
	(40%)	(<LOD ^a^–109)	(0%)	(- ^c^)	(0%)	(<LOD ^a^)
Etiopia	5/5	181	2/5	<LOD ^b^	4/5	84
	(100%)	(118–1620)	(40%)	(<LOD ^b^–30)	(80%)	(<LOD ^a^–108)
Antiqua	3/4	119.5	2/4	12.25	3/5	87
	(75%)	(<LOD ^a^–1467)	(50 %)	(<LOD ^b^–55)	(60%)	(<LOD ^a^–306)
Falaria	2/4	67.5	1/4	<LOD ^b^	0/4	<LOD ^a^
	(50%)	(<LOD ^a^–2215)	(25%)	(<LOD ^b^–41)	(0%)	(<LOD ^a^)
Eritrea	5/5	109	2/5	<LOD ^b^	3/5	123
	(100%)	(57–138)	(40%)	(<LOD ^b^–30)	(60%)	(<LOD ^a^–367)

^a^ Limit of detection (LOD) in blood samples = 50 ng/L. ^b^ Limit of detection (LOD) in milk samples = 15 ng/L; ^c^–not assessable.

## Supplementary Materials

The following are available online at https://www.mdpi.com/2072-6651/12/12/758/s1, Table S1: Ochratoxin A (OTA) levels in blood and milk samples collected from Antiqua and relevant foal after delivery. Table S2: Ochratoxin A (OTA) levels obtained by ELISA and HPLC (used as confirmatory method) in a subset of randomly selected blood samples (*n* = 17) of Antiqua and foals.
