# Supplementary material for: Natural Occurrence of Ochratoxin A in Blood and Milk Samples from Jennies and Their Foals after Delivery
Source: Toxins (Basel). 2020 Dec 1;12(12):758. doi: 10.3390/toxins12120758 (PMC7759822; doi:10.3390/toxins12120758)
Supplement: Supplementary file 1 [file toxins-12-00758-s001.pdf]

Supplementary Materials: Natural Occurrence of Ochratoxin A in Blood and Milk Samples from Jennies and Their Foals after Delivery

Vincenzo Lippolis, Shafaq Asif, Michelangelo Pascale, Salvatore Cervellieri, Erminia Mancini, Angelo Peli, Ippolito De Amicis, Domenico Robbe and Fiorenza Minervini

Table S1. Ochratoxin A (OTA) levels in blood and milk samples collected from jennies and relevant foal after delivery.

	**Jenny's Blood Samples**		**Foal's Blood Samples**	**Milk Samples**
Jenny name	Sampling date (month/day)	OTA content (ng/L)	OTA content (ng/L)	OTA content (ng/L)
Adelaide	April 3 (delivery)	52	<LOD[a]	<LOD[b]
	April 12	<LOD	110	<LOD
	April 21	<LOD	<LOD	<LOD
	May 1	1200	135	17
	May 15	1800	4034	43
	July 27	55	-[c]	-[c]
Francisca	April 15 (delivery)	97	<LOD	<LOD
	April 30	1700	160	35
	May 15	6000	600	80
	May 30	1500	140	20
	June 30	<LOD	145	<LOD
Gaia	June 18 (delivery)	109	<LOD	<LOD
	July 3	<LOD	<LOD	<LOD
	July 27	<LOD	<LOD	<LOD
	August 2	<LOD	<LOD	<LOD
	August 17	63	<LOD	<LOD
Etiopia	May 1 (delivery)	139	<LOD	<LOD
	May 16	1600	105	30
	May 31	860	80	20
	June 15	180	110	<LOD
	June 29	118	85	<LOD
Antiqa	April 1 (delivery)	<LOD	<LOD	-[c]
	April 12	80	<LOD	<LOD
	April 25	1470	190	55
	May 10	160	305	17
	May 24	-[c]	85	<LOD
Falaria	May 31 (delivery)	2215	<LOD	40
	June 15	110	<LOD	<LOD
	June 30	<LOD	<LOD	<LOD
	July 31	<LOD	<LOD	<LOD
Eritrea	July 13 (delivery)	79	<LOD	<LOD
	July 27	120	125	30
	August 11	57	<LOD	17
	August 27	140	140	<LOD
	September 10	110	370	<LOD

[a] Limit of detection (LOD) in blood samples = 50 ng/L. [b] Limit of detection (LOD) in milk samples = 15 ng/L.
[c] not collected.

Table S2. Ochratoxin A (OTA) levels obtained by ELISA and HPLC (used as confirmatory method) in a subset of randomly selected blood samples (*n* = 17) of jennies and foals.

		**Jenny's Blood Samples**		**Foal's Blood Samples**	
Jenny	Sampling date (month/day)	ELISA OTA content (ng/L)	HPLC OTA content (ng/L)	ELISA OTA content (ng/L)	HPLC OTA content (ng/L)
Adelaide	March 25 (before delivery)	<LOD [a]	<LOD [a]	- [b]	-
	April 12	<LOD	90	-	-
	May 15	-	-	4034	3700
Francisca	April 15 (delivery)	-	-	<LOD	<LOD
	April 30	1700	2400	-	-
	May 15	6000	7800	600	760
	May 30	1500	1700	-	-
	June 30	<LOD	<LOD	-	-
Etiopia	May 16	1600	1750	-	-
Antica	April 12	-	-	<LOD	<LOD
	April 15	1470	1550	-	-
	May 10	-	-	305	210
Falaria	May 21 (before delivery)	<LOD	<LOD	-	-
	May 31 (delivery)	2215	2200	-	-
Eritrea	July 13 (delivery)	-	-	<LOD	<LOD
	September 10	-	-	370	320

[a] Limit of detection (LOD) = 50 ng/L. [b] not confirmed by HPLC.
